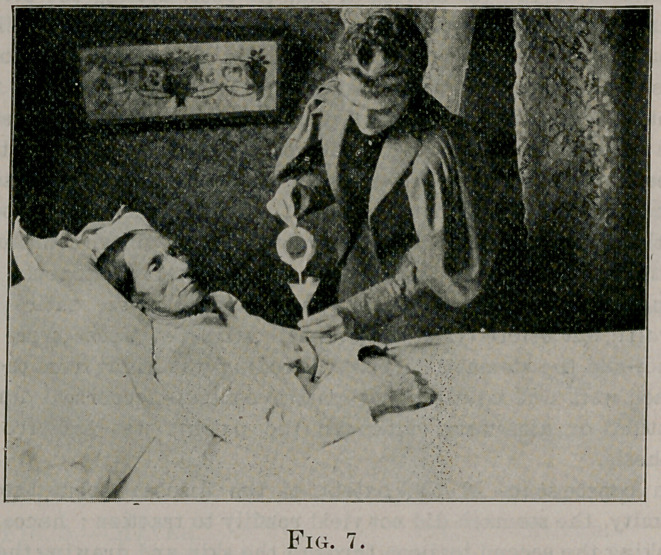# Modern Gastrostomy for Stricture of the Esophagus—with Report of a Case

**Published:** 1896-09

**Authors:** Lewis S. McMurtry

**Affiliations:** Louisville, Ky.; Professor of Gynecology and Abdominal Surgery in the Hospital College of Medicine, Louisville; Surgeon-in-Charge of the Jennie Casseday Infirmary for Women; Fellow of the American Association of Obstetricians and Gynecologists, etc., etc. 231 West Chestnut Street


BUFFALO MEDICAL JOURNAL.

Vol. XXXVI. SEPTEMBER, 1896. No. 2.

### Original Communications.

### MODERN GASTROSTOMY FOR STRICTURE OF THE ESOPHAGUS—WITH REPORT OF A CASE.

By LEWIS S. McMURTRY, A. M., M. D., of Louisville, Ky.,
Professor of Gynecology and Abdominal Surgery in the Hospital College of Medicine, Louisville; Surgeon-in-Charge of the Jennie Casseday Infirmary for Women; Fellow of the American Association of Obstetricians and Gynecologists, etc., etc.

CONSPICUOUS among the achievements of modern abdominal surgery are the great improvements in the surgery of the gastro-intestinal tract. Not only have many altogether new operations been devised and established, but some old operations have been improved and restored to favor by improved methods and a perfected technique. Among these latter is the operation of gastrostomy, whereby a gastric fistula is provided in cases of increasing cicatricial or cancerous stricture of the esophagus, through which the patient may be fed and spared the pangs of starvation.

The old method of direct incision and stitching the viscus to the margins of the parietal incision was practically rejected by the profession and is now abandoned. When the operation, as then done, succeeded, the result was far from satisfactory. The trouble was in keeping the fistulous tract tightly adjusted to the tube. Under the old method the subsequent regurgitation and leakage through the tube deprived the patient of the desired advantage and inflicted additional discomfort. The food poured into the stomach would flow back through and alongside the tube, so that the patient was not nourished ; and the uncleanliness and excoriation consequent upon this condition were painful and annoying. And to this the danger of the operation itself was added, a large proportion of cases terminating fatally from septic peritonitis and pneumonia following the operation.

The skill and ingenuity of modern surgeons have now provided several methods of performing gastrostomy which are free from these objections. Moreover, modern gastrostomy is now perfected to such an extent that no patient with stenosis of the esophagus need die of starvation, while the operation itself involves but trifling danger when properly performed. These improved methods have placed the indications for gastrostomy upon an entirely new basis. It is no longer admissible to let the patient with malignant or cicatricial stenosis of the esophagus go until fluids cannot pass into the stomach ; or, as sometimes, until rectal alimentation will no longer suffice. As Meyer puts it, in cases of cancer of the esophagus, a gastric fistula should be established as soon as the scales show a steady decrease in weight ; in cases of a burn of the esophagus, gastrostomy and timely dilatation may prevent incurable conditions.

Gastrostomy, as formerly performed, consisted of a three-inch incision made parallel with the border of the ribs on the left side, from near the median line down to the eighth costal cartilage. The parietes were divided, including the peritoneum, and the anterior wall of the stomach attached by sutures to the margins of the abdominal incision. An area of the gastric surface, about one and one-half inches long and one inch wide, was attached by sutures, and a loose tamponade applied. From three to five days thereafter the stomach was incised, a small opening being made and a tube inserted. In the meantime the patient was maintained by rectal alimentation. This method is known as Fenger's, the operation having been modified and practised, in 1853, by C. E. F. Fenger, of Copenhagen.

As already stated, the objection to this primitive method of gastrostomy is the almost universal leakage around the tube, rendering the operation functionally inefficient. In consequence, the operation of gastrostomy fell into disfavor and Fenger's method is now obsolete.

Of the several methods recently devised, and which overcame all former difficulties and objections, I shall describe only two, since I am sure that one or the other of these operations will meet the requirements of all cases.[1]

*Witzel's Method.*—This procedure, devised and practised by Oscar Witzel, was published in 1891, and is in many particulars preferable to all other methods. As a primary operation in cases

1. I have omitted detailed description of Von Hacker's method, which, on account of ease and rapidity of performance, is suited for extreme cases unable to bear other operations. Its field is limited to such cases ; its functional results are not equal to the two operations described. Hahn's method is likewise omitted, being inferior to the two methods described.

of burn of the esophagus, wherein timely dilatation of the cicatricial construction is applied, it is superior to every other method. It has the double advantage of preventing all leakage and of closing spontaneously when the tube is removed and left out. At the same time, it is more difficult to perform than the other method to be described ; has an element of immediate danger in opening the viscus prior to stitching the peritoneum, and requires the tube to be worn under the constant supervision of the surgeon, so that readjustment may be promptly made if the tube should be extruded. The operation is thus performed: Fenger's oblique incision is first

FIG. 1.—MEYER.

FIG. 2—MEYER.

made through the skin and cellular tissue. A longitudinal incision is then made in the sheath of the rectus muscle and blunt separation made of the fibers of the rectus and transversalis muscles parallel to their course. While these are held aside by blunt hooks, the peritoneum is divided obliquely. The stomach is drawn out, packed around with gauze, and incised near the fundus sufficiently to admit a quarter-inch diameter rubber tube. The tube is infolded by Lembert sutures to the extent of about one and one-half inches upward in the oblique direction of the external incision, also extending the line of sutures half an inch below the opening into stomach. (See Fig. 1.) When the sutures are tied, the tube will be infolded, as shown in Figure 2. The stomach is then dropped back and the peritoneum shut off by suturing all around the operative area to the parietal peritoneum. The abdominal incision down to the peritoneum is then closed with silkworm-gut sutures up to the upper angle where a snug opening is left for the tube. When the blunt hooks are removed from the separated muscles, it will be observed that the fibers of these muscles securely clamp the tube. In cases where this method has been applied by Witzel, Mikulicz, and Meyer, the fistula has remained pervious for months without regurgitation or leakage, and in one case closed spontaneously when the tube was left out.

*Ssabanejew-Frank Method.*—According to Dr. Willy Meyer, of New York, to whose writings I am greatly indebted, as well as

FIG. 3.—MEYER—CONE OF STOMACH DRAWN THROUGH INCISION AND ITS BASE SUTURED TO PERITONEUM.

for the illustrations of this paper, Ssabanejew, of Odessa, devised and performed the valuable method next to be described, in 1890. In 1892, R. Frank, assistant to Albert, in Vienna, devised independently and without knowledge of Ssabanejew's work, practised the same procedure.

This operation is begun with Fenger's incision. The muscles having been bluntly separated in the direction of their fibers the peritoneum is opened. The stomach is drawn forward and a silk sling is passed through the outer coats at a point near the fundus, so as to draw outside a cone of about one and one-half inches. (See Fig. 3.) The edges of the parietal peritoneum are now sutured to the serous coat of the stomach around this cone, so as to shut off the general peritoneum. Frank advises that the sutures include the muscles also. A second incision is now made about one inch above the border of the ribs, parallel to the first incision, and about one-half to three-quarters of an inch in length. This incision divides skin only. The skin between the two incisions is raised from the cartilage by blunt dissection and the sling thread passed underneath and out the smaller incision, drawing the cone of stomach out. (See Fig. 4.) The first incision is now closed with silkworm-gut sutures. The projecting apex of the cone of the stomach is now incised for about half an inch and its mucous membrane sutured to the skin. (See Fig. 5.)

FIG. 4.—MEYER.

Feeding may be instituted immediately. The following dia gram (Fig. 6), also from Meyer's paper, illustrates the indirect and valvular opening into the stomach. The pressure upon the fistulous canal between the skin and costal cartilage, as well as by muscular compression, has proved quite as effective in preventing leakage as in the method of Witzel. In the case appended to this article I depended altogether upon the muscular force for the necessary compression, and with most satisfactory results. The management of the tube after operation by this method is very simple. The tube need not be constantly worn and the patient can, in most instances, feed and care for himself or herself.

This operation has now been done by Ssabanejew and Frank, four cases each ; Meyer,[1] three cases ; Winslow,[2] two cases ; Max Stern,[3] one case ; Thomas S. K. Morton,[3] one case ; Hupp,[4] one case; Edward Martin,[5] one case; and Finney, one case. In all these cases the results were quite satisfactory, so far as the working of the fistulæ was concerned, and the method merits the highest commendation. In one of Winslow's cases, wherein the operation was on account of cicatricial stenosis, the artificial opening acted efficiently during four months while instrumental treatment was carried on and the caliber of the esophagus restored. The edges of the fistula were then pared and sutured, and permanent closure was promptly obtained.

FIG. 5.—MEYER.

FIG. 6.—MEYER.

REPORT OF CASE.—Mrs. R., aged 70, was admitted to the infirmary under my care, January 29, 1896. I had visited her the preceding day at her home and her condition was indeed distressing and pitiable. She was emaciated, her features were pinched, and her efforts to swallow soup, milk and water were constant and persistent. A small quantity of fluid would be retained in the sacculated portion of the esophagus above the stricture, only to be rejected in a few minutes. She was suffering intensely from the pangs of thirst and hunger. I at once instituted systematic rectal alimentation and had her removed to the infirmary the following day. Sounding revealed impermeable stricture of the cardiac end of the esophagus, undoubtedly the result of malignant disease.

The following day I did gastrostomy after the Ssabanejew-Frank method above described, with a slight modification. The parietal incision was made parallel to and three-quarters of an inch below the costal arch of the left side and to the outer side of the left rectus muscle. The incision was three and one-half inches in length and the peritoneum was exposed and divided to the same extent. The stomach was readily identified and drawn into the incision. A silkworm-gut suture was passed through the serous and muscular coats, so that a cone of the stomach wall could be drawn out through the incision. Passing a finger along the upper surface of the viscus, toward the esophageal entrance, an indurated mass could be felt posteriorly, which interfered with free traction. The traction-suture was placed as near the esophageal entrance as necessary traction would permit. While the cone was firmly drawn into the incision a continuous suture of chromicised catgut was carried around its base, uniting the serous and muscular coats of the stomach to the parietal peritoneum. Silkworm-gut sutures were then placed to close the abdominal incision, these sutures being carried down to, but not through, the peritoneum, with the exception of two now to be mentioned. At the upper and lower angles of the incision the silkworm-gut sutures were carried through the parietal peritoneum and also through the serous and muscular coats of the stomach. These strong sutures are of essential importance to firmly hold the viscus in place and avoid undue traction on the continuous catgut suture, uniting the stomach to the parietal peritoneum. After placing the silkworm-gut sutures (passing through the entire abdominal wall), but before tying them, small strips of gauze were packed around, and the stomach opened. Bleeding was slight from the incised stomach wall, and no outflow of gastric contents occurred during the operation or afterward, although the patient was sick from ether anesthesia.

In consequence of the extent of the disease about the cardiac extremity, the stomach did not yield readily to traction ; hence, instead of making the second incision through the skin and drawing the cone of stomach through, I sutured the incision so that the tube would emerge from the opposite end of the incision from the opening in the stomach. That is, the stomach was opened opposite the lower angle of the incision and the tube was sutured over in the parietes and allowed to emerge from the upper angle of the incision. Since immediate union took place, the same valvular course was obtained for the tract of the tube, and retention was effected.

No shock followed the operation and barring some nausea from the anesthetic the patient was quite as comfortable immediately after the operation as before. The pulse was normal—72 to 76—throughout. Feeding was instituted the following day, giving liquids through the tube. The quantity given at first was small and was gradually increased. After the third day the patient was encouraged to sit up in bed, half reclining on pillows, in order to prevent pneumonia, which is so often a fatal complication after this operation. There was slight regurgitation through the tube at first, but this soon ceased, and gradually increasing quantities of food and water were retained. There was no leakage at any time alongside the tube. The incision united throughout by immediate union.

At the end of two weeks the patient was up and about her room. She gained strength quickly and the increase of flesh, as indicated by weight, was marked and steady. The torture of thirst and hunger was relieved immediately. She had much comfort from chewing tender pieces of beefsteak and chicken, thereby gratifying the sense of taste. Owing to the relief from irritation (caused by constant efforts at swallowing before operation), the stricture permitted some fluid to pass dur-

FIG. 7.

ing attempts at swallowing. At the end of the third week the patient returned to her home, being quite able, with the assistance of her niece, to feed herself. The photograph (Fig. 7) was taken at this time.

For four months the patient got on very well, when the systemic effects of the disease were manifested by emaciation and marked cachexia. She failed thereafter and died during the last week in May. The gastric fistula remained tight throughout, and the relief and comfort afforded by the operation were incalculable.

1. American Journal of Medical Sciences, October, 1894.
2. Annals of Surgery, May, 1895.
3. Morton's paper, Medical News, January 25, 1896, page 87.
3. Medical News, January 25, 1896, page 86.
4. New York Medical Journal.
5. Annals of Surgery, March, 1896.
6. Bulletin Johns Hopkins Hospital, February, 1895.

231 WEST CHESTNUT STREET.

## Figures and Tables

**Fig. 1. f1:**
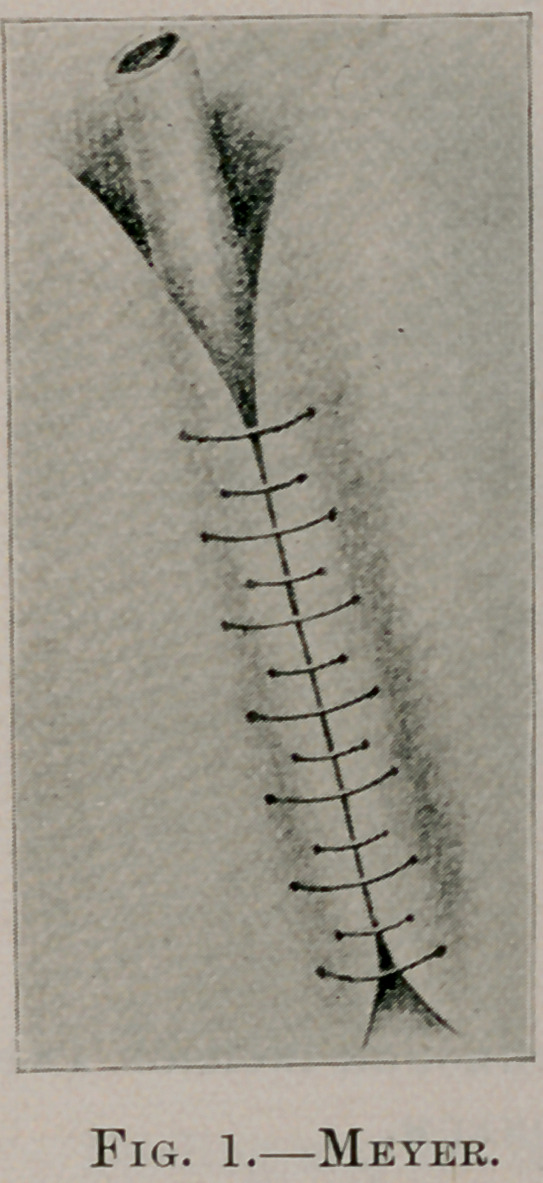


**Fig. 2. f2:**
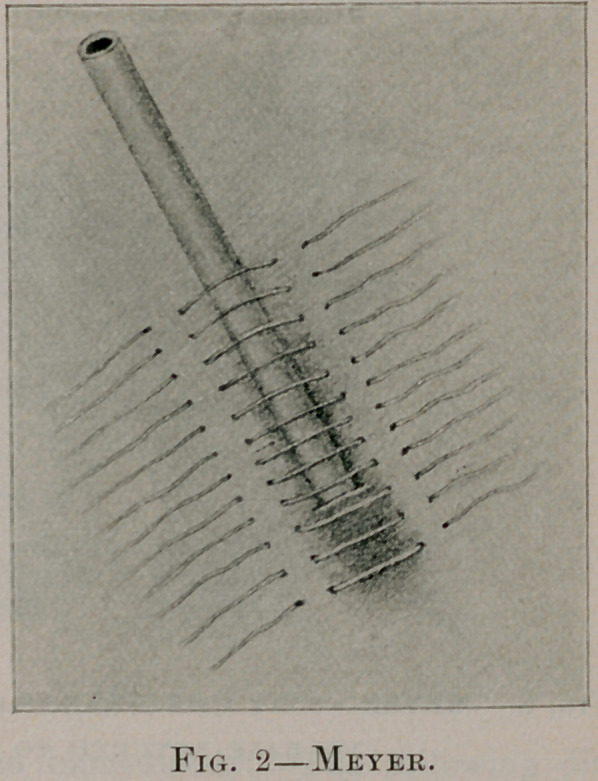


**Fig. 3. f3:**
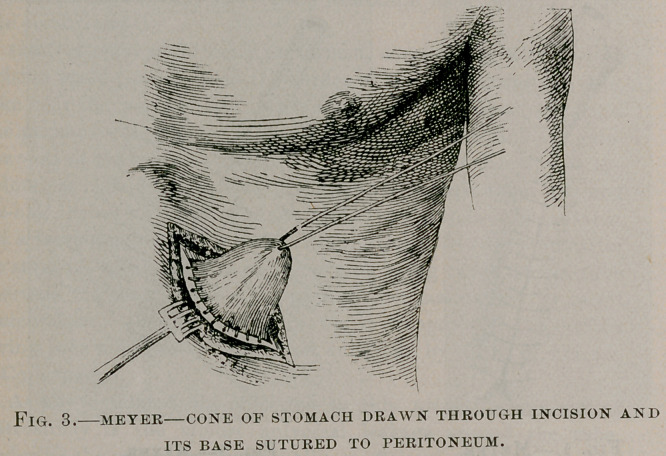


**Fig. 4. f4:**
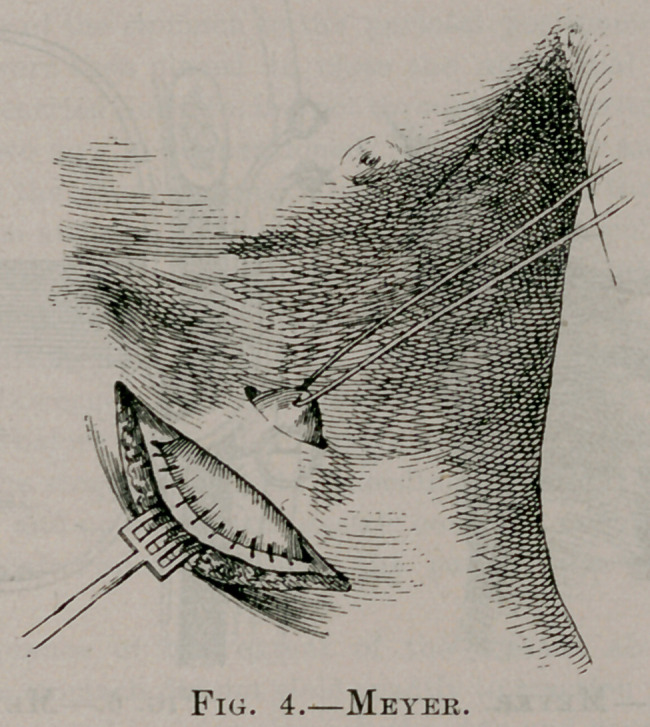


**Fig. 5. f5:**
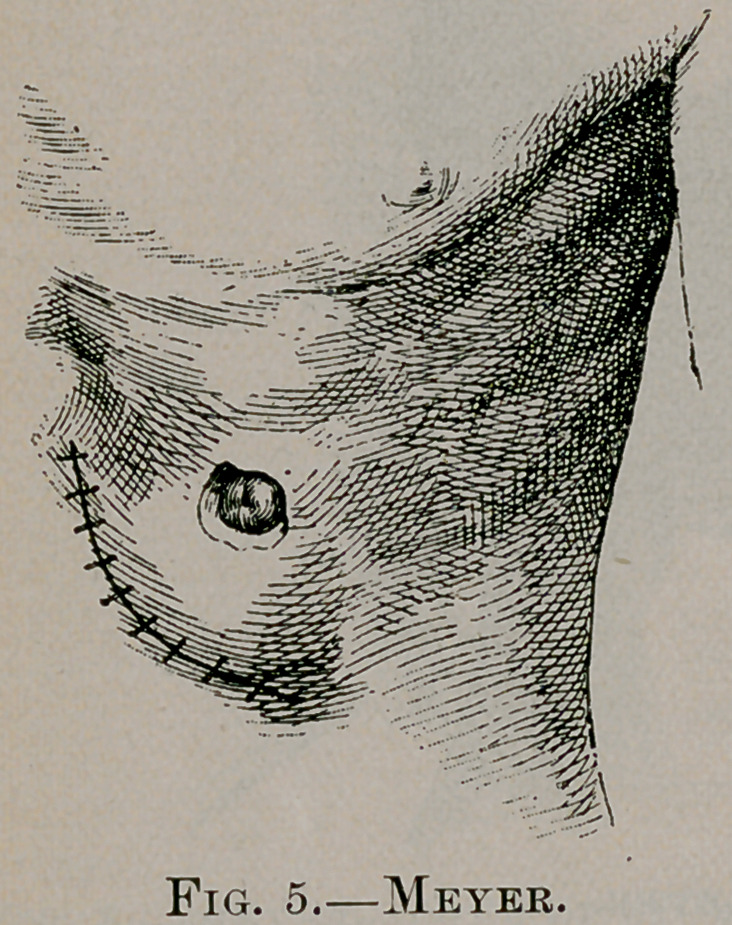


**Fig. 6. f6:**
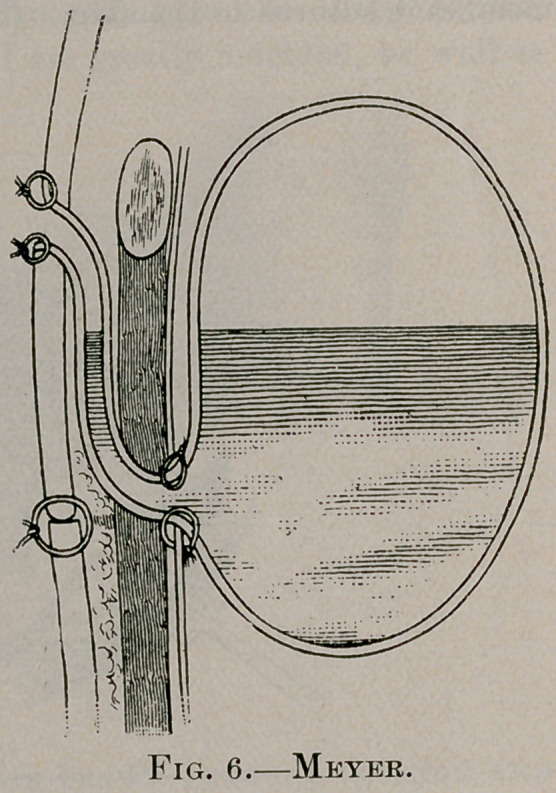


**Fig. 7. f7:**